# Complete Genome Sequence of the Model Oleaginous Alga *Nannochloropsis gaditana* CCMP1894

**DOI:** 10.1128/genomeA.01448-17

**Published:** 2018-02-15

**Authors:** Ariel S. Schwartz, Rob Brown, Imad Ajjawi, Jay McCarren, Suzan Atilla, Nick Bauman, Toby H. Richardson

**Affiliations:** a Synthetic Genomics, La Jolla, California, USA

## Abstract

The model oleaginous alga *Nannochloropsis gaditana* was completely sequenced using a combination of optical mapping and next-generation sequencing technologies to generate one of the most complete eukaryotic genomes published to date. The assembled genome is 30.7 Mb long.

## GENOME ANNOUNCEMENT

As the world moves away from fossil fuels to more sustainable and renewable energy sources, photosynthetic algae have shown great promise as a potential alternative liquid fuel. *Nannochloropsis gaditana* and other members of the genus *Nannochloropsis* have been extensively studied because of their natural ability to make lipids (1–3), and there have been several publications of draft whole-genome sequences, transcriptomes, and organelle genomes for this species (4–10). Recently, we published a doubling of lipid productivity in this species by decreasing the expression levels of a single transcription factor (11).

*Nannochloropsis gaditana* CCMP1894 was obtained from CCMP, now known as the National Center for Marine Algae and Microbiota (https://ncma.bigelow.org/), cell sorted to a single cell, and propagated to produce a clonal population. The strain was cultivated as described previously (9), and DNA was isolated by grinding with liquid nitrogen, followed by a general phenol-chloroform clean-up procedure. The genome was sequenced using PacBio single-molecule real-time (SMRT) sequencing technology. In addition, optical mapping (12) was used to generate chromosome size scaffolds and to aid in gap filling. Several PacBio SMRTbell libraries were made and sequenced on a total of 26 SMRT cells on the PacBio RS II platform, for a total of 8.86 Gb. The PacBio data were assembled using Hierarchical Genome Assembly Process 2 (HGAP2) into 92 nuclear contigs (after removal of 20 contigs, including the plastidial and mitochondrial contigs, as well as contigs determined to originate from bacterial contamination), and the resulting contig sequences were polished with Quiver. The polished assembled contigs were then mapped and assigned to chromosomes using an optical map obtained from OpGen (Gaithersburg, MD) as a reference. Out of the 92 nuclear contigs, 39 contigs were uniquely assigned to chromosomal positions. Further manual finishing resulted in the closure of 5 of the 9 remaining gaps.

The final assembly was 30.7 Mb long, and it consisted of 30 chromosomal scaffolds (29.98 Mb; 97.6%) and 53 unplaced contigs (725 kb; 2.4%). Out of the 30 chromosomal scaffolds, 23 scaffolds included both telomeric sequences and no gaps, 4 scaffolds included both telomeric sequences and a single gap each, and 3 scaffolds included one of the two telomeric sequences with no additional gaps. The earlier draft genome sequences of *Nannochloropsis* species have been useful for understanding the basic metabolic machinery and genes involved in important processes, such as lipid metabolism. However, these genomes were highly fragmented and are not suitable for understanding chromosome architecture or to effectively engineer chromosomes, and they almost certainly do not annotate a number of functional genes (11). This near-complete assembly will enable future studies to better understand lipid metabolism, carbon partitioning, and photosynthetic efficiency and the ability to manipulate chromosomes within this model oleaginous alga (13).

### Accession number(s).

This whole-genome shotgun project has been deposited at DDBJ/ENA/GenBank under the accession number PEIC00000000.

## References

[1] Radakovits R, Jinkerson RE, Fuerstenberg SI, Tae H, Settlage RE, Boore JL, Posewitz MC 2012 Draft genome sequence and genetic transformation of the oleaginous alga *Nannochloropsis gaditana*. Nat Commun 3:686. doi:10.1038/ncomms1688. 22353717 PMC3293424

[2] Ma XN, Chen TP, Yang B, Liu J, Chen F 2016 Lipid production from *Nannochloropsis*. Mar Drugs 14:61–78. doi:10.3390/md14040061. PMC4849066 27023568

[3] Griffiths MJ, Harrison STL 2009 Lipid productivity as a key characteristic for choosing algal species for biodiesel production. J Appl Phycol 21:493–507. doi:10.1007/s10811-008-9392-7.

[4] Wang D, Ning K, Li J, Hu J, Han D, Wang H, Zeng X, Jing X, Zhou Q, Su X, Chang X, Wang A, Wang W, Jia J, Wei L, Xin Y, Qiao Y, Huang R, Chen J, Han B, Yoon K, Hill RT, Zohar Y, Chen F, Hu Q, Xu J 2014 *Nannochloropsis* genomes reveal evolution of microalgal oleaginous traits. PLoS Genet 10:e1004094-13. doi:10.1371/journal.pgen.1004094. 24415958 PMC3886936

[5] Carpinelli EC, Telatin A, Vitulo N, Forcato C, D’angelo M, Schiavon R, Vezzi A, Giacometti GM, Morosinotto T, Valle G 2014 Chromosome scale genome assembly and transcriptome profiling of *Nannochloropsis gaditana* in nitrogen depletion. Mol Plant 7:323–335. doi:10.1093/mp/sst120. 23966634

[6] Vieler A, Wu G, Tsai CH, Bullard B, Cornish AJ, Harvey C, Reca IB, Thornburg C, Achawanantakum R, Buehl CJ, Campbell MS, Cavalier D, Childs KL, Clark TJ, Deshpande R, Erickson E, Ferguson AA, Handee W, Kong Q, Li X, Liu B, Lundback S, Peng C, Roston RL, Sanjaya, Simpson JP, Terbush A, Warakanont J, Zauner S, Farre EM, Hegg EL, Jiang N, Kuo M-H, Lu Y, Niyogi KK, Ohlrogge J, Osteryoung KW, Schachar-Hill Y, Sears BB, Sun Y, Takahashi H, Yandell M, Shiu SH, Benning C 2012 Genome, functional gene annotation and nuclear transformation of the heterokont oleaginous alga *Nannochloropsis oceanica* CCMP1779. PLoS Genet 8:e1003064. doi:10.1371/journal.pgen.1003064. 23166516 PMC3499364

[7] Kehou P, Junjie Q, Si L, Wenkui D, Baohua Z, Yuanchun J, Wengdong Y, Guanpin Y, Dongfang L 2011 Nuclear monoploidy and asexual propagation of *Nannochloropsis oceanica* (Eustigmatophyceae) as revealed by its genome sequence. J Phycol 47:1425–1432. doi:10.1111/j.1529-8817.2011.01057.x. 27020366

[8] Jinkerson RE, Radakovits R, Posewitz MC 2013 Genomic insights from the oleaginous model alga *Nannochloropsis gaditana*. Bioengineered 4:37–43. doi:10.4161/bioe.21880. 22922732 PMC3566019

[9] Wei L, Xin Y, Wang D, Jing X, Zhou Q, Su X, Jia J, Ning K, Chen F, Hu Q, Xu J 2013 *Nannochloropsis* plastid and mitochondrial phylogenomes reveal organelle diversification mechanism and intragenus phylotyping strategy in microalgae. BMC Genomics 14:534. doi:10.1186/1471-2164-14-534. 23915326 PMC3750441

[10] Starkenburg SR, Kwon KJ, Jha RK, McKay C, Jacobs M, Chertkov O, Twary S, Rocap G, Cattolico RA 2014 A pangenomic analysis of *Nannochloropsis* organellar genomes reveals novel genetic variations in key metabolic genes. BMC Genomics 15:212–232. doi:10.1186/1471-2164-15-212. 24646409 PMC3999925

[11] Ajjawi I, Verruto J, Aqui M, Soriaga LB, Coppersmith J, Kwok K, Peach L, Orchard E, Kalb R, Xu W, Carlson TJ, Francis K, Konigsfeld K, Bartalis J, Schultz A, Lambert W, Schwartz AS, Brown R, Moellering ER 2017 Lipid production in *Nannochloropsis gaditana* is doubled by decreasing expression of a single transcriptional regulator. Nat Biotechnol 35:647–652. doi:10.1038/nbt.3865. 28628130

[12] Schwartz DC, Li X, Hernandez L, Ramnarain SP, Huff EJ, Wang YK 1993 Ordered restriction maps of *Saccharomyces cerevisiae* chromosomes constructed by optical mapping. Science 262:110–114. doi:10.1126/science.8211116. 8211116

[13] Li WC, Huang CH, Chen CL, Chuang YC, Tung SY, Wang TF 2017 *Trichoderma reesei* complete genome sequence, repeat induced point mutation, and partitioning of CAZyme gene clusters. Biotechnol Biofuels 10:170–189. doi:10.1186/s13068-017-0825-x. 28690679 PMC5496416

